# Exploring the Intersection Between Informal Carers’ Experiences, Digital Poverty and Poor Socioeconomic Status, Protocol

**DOI:** 10.1192/bjo.2025.10201

**Published:** 2025-06-20

**Authors:** Peter Sunny Blaney, Oladayo Bifarin, Mikolaj Zarzycki, Pooja Saini, Rosanna Cousins

**Affiliations:** ^1^Liverpool John Moores University, Liverpool, United Kingdom; ^2^Liverpool Hope University, Liverpool, United Kingdom

## Abstract

**Aims:** In 2021–2022, 10.5% of UK citizens provided unpaid informal care, saving the government £162 bn annually. Many carers reside in high-deprivation areas, where access to appropriate health and social care services is limited. Previous studies indicate that carers are more prone to depression, anxiety, and physical symptoms, and these negative outcomes are higher among socio-economically disadvantaged carers. The shift of some health and social care services online, combined with ‘digital poverty’ (having no suitable electronic devices with Internet access or limited access or skills concerning the Internet), may exacerbate difficulties with accessing health and social care support, potentially increasing unmet needs and burdens among socioeconomically disadvantaged carers. The aim is to understand how informal carers with marginalised socioeconomic status (SES) access existing health and social care services and how this impacts their mental health. The second aim of the project is to explore how potential digital poverty may shape a carer’s mental health outcomes.

**Methods:** A systematic literature review will identify barriers and facilitators of accessing health and social care by informal carers, the impact of access/non-access on mental health, stratified by carer SES and care-recipient’s health conditions. Followed by a qualitative photovoice study to explore carers’ experiences of accessing health and social care and the effects of digital poverty, analysed through critical discourse analysis. Thirdly a survey (N >300) examining how factors underpinning access to health and social care are related to informal carers’ mental health as moderated or mediated by the caregiver SES, carers’ perceptions of access to health and social care and of digital poverty analysed by structural equation modelling.

**Results:** We will identify if and how informal carers with a marginalised socioeconomic background access health and social care services. Which will allow us to develop an evidence-based health promotion model.

**Conclusion:** This study will offer us a unique opportunity to develop an evidence-based health promotion model for these carers that shows how to mitigate existing pathways of health inequalities. Based on key findings, recommendations will be generated and shared with researchers, clinicians, and policymakers via academic publications, conferences, exhibitions of carers’ photographs, and carer forums with NHS Trust(s).

